# Intra-esophageal whitish mass – a challenging diagnosis

**DOI:** 10.1186/s12876-015-0335-x

**Published:** 2015-08-19

**Authors:** Lidia Ciobanu, Oliviu Pascu, Marcel Tantau, Oana Pinzariu, Bogdan Furnea, Emil Botan, Marian Taulescu

**Affiliations:** 1 Regional Institute of Gastroenterology and Hepatology, University of Medicine and Pharmacy, Croitorilor Street 19-21, Cluj-Napoca, 400162 Romania; 2 Emergency Clinic Country Hospital, Cluj-Napoca, 400006 Romania; 3 Regional Institute of Gastroenterology and Hepatology, Cluj-Napoca, 400162 Romania; 4 Department of Pathology, Emergency Clinic Country Hospital, Cluj-Napoca, 400006 Romania; 5 Department of Pathology, Faculty of Veterinary Medicine, University of Agricultural Science and Veterinary Medicine, Cluj-Napoca, 400372 Romania

## Abstract

**Background:**

Whitish intraluminal esophageal masses might represent the endoscopic feature of a bezoar or a pedunculated tumor, most likely a fibrovascular polyp, without exclusion of other mesenchymal tumors (leiomyoma, lipoma, gastrointestinal stromal tumor, leiomyosarcoma, granular cell tumor). If a process of dystrophic calcification is also encountered the differential diagnosis can be a challenge even after histological analysis, as it is highlighted by our case.

**Case presentation:**

A 65-year-old female whom took lactate calcium tablets for 5 years presented with progressive dysphagia. A whitish esophageal mass with an appearance of a pharmacobezoar was detected at esophagoscopy. A pedunculated tumor was considered in the differential diagnosis, but the imagistic studies ruled out a pedicle. This intraluminal esophageal mass highly suggestive for a pharmacobezoar was endoscopically removed. The challenge of correct diagnosis was raised by histological examination performed after immersion into trichloracetic acid for decalcification. The identification of hyaline fibrous tissue, with numerous crystalline basophils deposits of minerals, rare fibrocytes and very few vessels brought in discussion a mesenchymal originating mass, most likely a fibrovascular polyp, even the pedicle was not detected.

**Conclusion:**

Based on our challenging and difficult to diagnose case we proposed an uncommon evolution: auto-amputation and calcification of an esophageal mesenchymal originating tumor (most likely a fibrovascular polyp).

## Background

Esophageal intra-luminal whitish mass might represent the endoscopic appearance of a bezoar or a fibrovascular polyp, without exclusion of other mesenchymal tumors with intraluminal polypoid aspect (leiomyosarcoma, gastrointestinal stromal tumor, leiomyoma).

Bezoars, retained concretions of indigestible foreign materials that accumulate and conglomerate are rarely seen in the esophagus. Esophageal pharmaco-bezoars are associated with structural or functional abnormalities of the esophagus in addition to specific medication: antihypertensive calcium blockers [1], clomipramine [2] or glucomannan (polysaccharide) [3]. Esophageal bezoars were reported in patients with enteral feeding, most of them also receiving sucralphate or aluminum hydroxide antacids, medication known to cause bezoars [4].

The pedunculated esophageal masses are frequently represented by fibrovascular polyps, considered tumor-like lesions [5, 6]. This lesion, unique to esophagus, developed from the upper esophagus; may be due to redundant folds that get pulled down by force of swallowing [7, 8]. It is defined as a polyp composed of a core of fibrous or fibro-adipose connective tissue and blood vessels covered by thickened but otherwise normal squamous epithelium [5, 6]. It is also called fibroma, fibrolipoma, fibromyxoma and may actually be an acquired malformation or hamartoma [7, 8]; it is not included in the mesenchymal tumors of the esophagus by World Health Organisation [9]. Autoamputation of polypoid lesions in the gastrointestinal tract is rare phenomenon, and it presumably occurs due to ischemic necrosis of the polyp by peristalsis-induced torsion or tension [10], being described for gastric polyps [11] and colonic lypoma [10, 12], not described for fibrovascular polyps of the esophagus.

The mesenchymal tumors (leiomyoma, lipoma, gastrointestinal stromal tumor, leiomiosarcoma, granular cell tumor, haemangioma) are defined as a group of nonepithelial tumors with variable histogenesis, including smooth muscle, stromal (Cajal) cells, fibroblastic/myofibroblastic, endothelial origin [9]. They develop as intramural nodules, frequently detected as submucosal lesions at endoscopy. They have different patterns of growth: leiomyoma frequently develop intramurally with mediastinal extension, but leiomyosarcoma might present as polypoid intraluminal masses [9, 13].

We present a challenging diagnosis regarding an intraluminal esophageal whitish mass, initially supposed to be a bezoar based on macroscopic appearance with the lack of a pedicle, but not sustained by histological analysis. The presence of hyaline fibrous tissue, with numerous crystalline basophils deposits of minerals, rare fibrocytes and very few vessels brought in discussion a mesenchymal originating mass, most likely a fibrovascular polyp, with an uncommon evolution: autoamputation and calcification.

## Case presentation

**Fig. 1:**
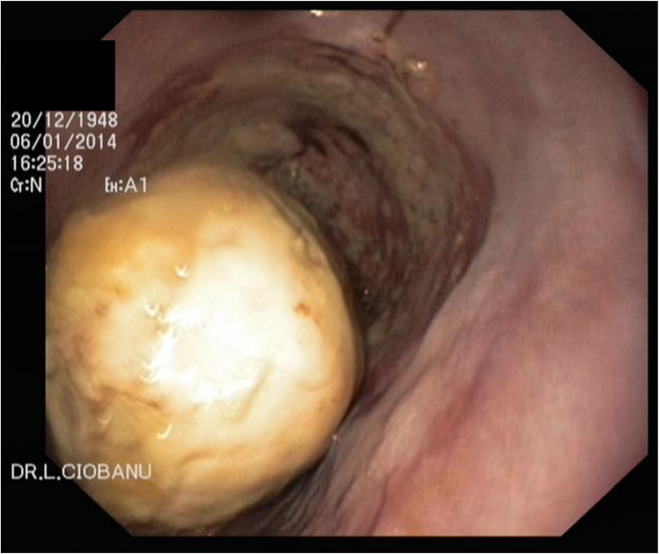
Upper gastrointestinal endoscopy: a 4 cm whitish-grey firm mass present in the middle esophagus

**Fig. 2:**
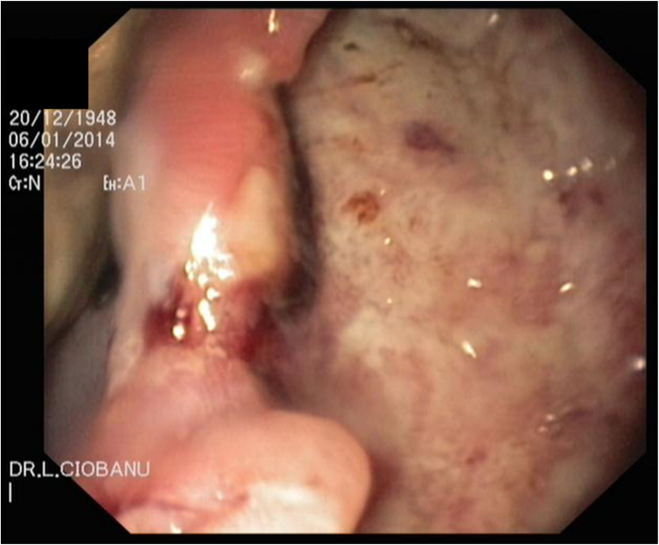
Upper gastrointestinal endoscopy: a semi-circumferential deep ulcer with irregular borders on the opposite mucosa to the intraluminal mass

**Fig. 3:**
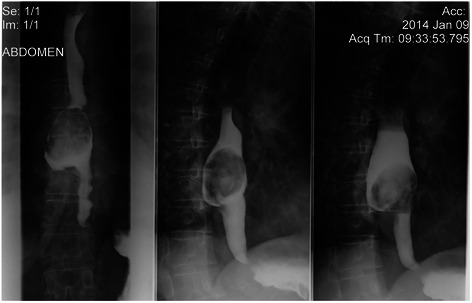
Upper gastrointestinal series with gastrografin: an ovoid lacunar image at the distal part of the esophagus esophageal, inhomogeneous, with calcifications and smooth contours. The esophageal lumen was enlarge with a diverticula development at the posterior wall

**Fig. 4:**
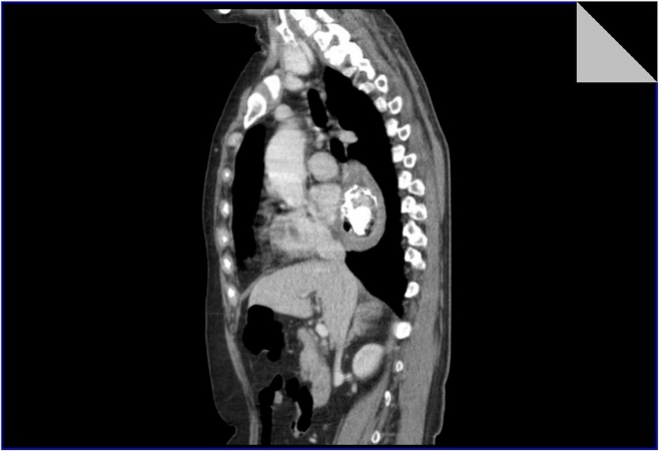
CT of the thorax excluded a pedunculated tumor, describing an intra-luminal calcified esophageal mass

A 65-year old female was admitted with progressive dysphagia for 2 months and 5 kg weight loss. Her past medical history was significant for osteoporosis treated with calcium lactate tablets, daily, for 5 years. Upper gastrointestinal endoscopy described a 4 cm whitish firm mass in the middle esophagus (Fig. 1) and a semi-circumferential deep ulcer with irregular borders on the opposite mucosa (Fig. 2). During endoscopy a pedicle was not identify by handling a polipectomy snare around the esophageal mass. Upper gastrointestinal series with gastrografin (Fig. 3) revealed an ovoid lacunar image at the distal part of the esophagus esophageal, inhomogeneous, with calcifications and smooth contours. During peristalsis the image was mobile and no pedicle was identified. The esophageal lumen was enlarged with a diverticula development at the posterior wall. Also computer tomography of the thorax excluded a pedunculated tumor, describing an intra-luminal calcified esophageal mass (Fig. 4). The biopsies obtained from the esophageal ulcerated mucosa revealed inflammatory cells, without malignancy. Based on these endoscopic and imagistic results a bezoar was supposed to have been developed in an esophageal diverticula, subsequently with ulcerated mucosa.

**Fig. 5:**
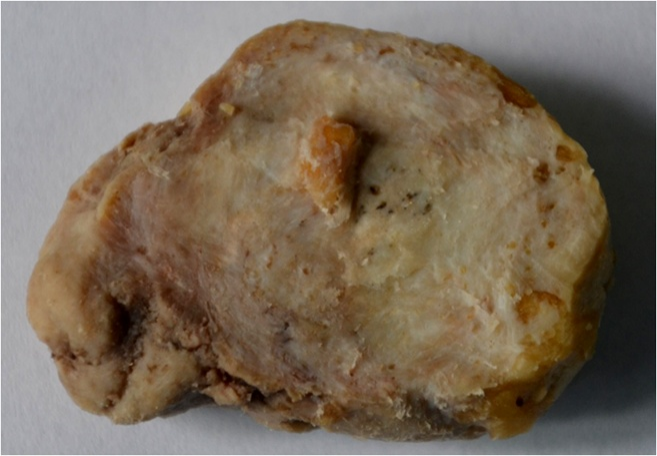
The cross section macroscopic appearance: a 4 cm, globular mass, heterogeneous, whitish, in places with yellow foci, most likely dystrophic calcification

**Fig. 6:**
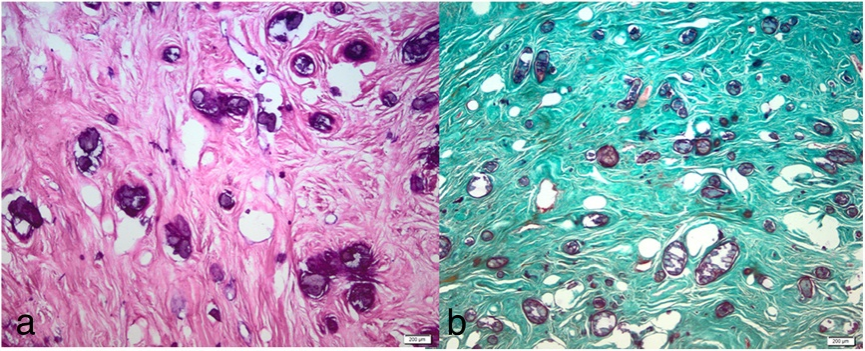
Microscopic examination (200 μm) revealed an acellular hyaline fibrous tissue (**a**), stained in green in Tricrom Mason (**b**), with numerous crystalline deposits, basophiles, and very few vessels

The esophageal mass was removed with an endoscopic snare in one piece, as the fragmentation was not physically possible. The macroscopic appearance revealed a 4 cm, globular mass, heterogeneous, dense, whitish, in places with harsh yellow foci, most likely dystrophic calcification. The macroscopic examination on cross section revealed a light gray aspect (fibrous appearance) that includes multiple harsh yellow-orange structures, difficult to section (Fig. 5). This mass was immersed into trichloracetic acid for decalcification. Microscopic examination revealed hyaline fibrous tissue (Fig. 6a), stained in green in Tricrom Mason (Fig. 6b), with numerous crystalline basophils deposits of minerals, rare fibrocytes and very few vessels. The presence of capillary structures, rare fibroblasts and collagen fibers brought in discussion a mesenchymal originating mass, most likely a fibrovascular polyp. A definitive histological diagnosis was not possible, as the pedicle was not identified, but the presence of the connective tissue suggested the previous presence of a pedicle into the lesion. The long term calcium tablets intake might explain the calcification process developed into the vascular-connective tissue, revealed on histology by the numerous crystalline basophils deposits of minerals.

One month later the patient was asymptomatic. The endoscopy did not revealed an enlarged esophagus, the appearance of the esophageal mucosa was normal (without ulcerations) and no diverticula was identified. No motility disturbances were found on esophageal manometry.

Based on the clinical course, the history of calcium lactate intake and histological appearance a diagnosis of an esophageal benign mesenchymal originating mass (most probably a fibrovascular polyp) auto-amputated and calcified was formulated.

## Discussion

After the first endoscopy a challenging differential diagnosis process was carried out.

The intra-luminal whitish mass might have represented a bezoar developed in an esophageal diverticula with secondary ulceration of the diverticular mucosa, a fibrovascular polyp or a mesenchymal tumor (leiomyosarcoma or gastrointestinal stromal tumor) presented as a polypoid intraluminal mass with decubitus lesions on the opposite mucosa. As a pedicle was not documented on endoscopy by hadling a polypectomy snare around the mass, on X series or on computer tomography scan, a bezoar was considered. Other arguments for this initial diagnosis were the history of calcium tablets intake and the calcified mass described by imagistic studies.

The calcium lactate tablets have not been associated to a pharmacobezoar development in the literature, but calcium polystyrene sulfonate, an exchange resin used to treat hyperkalemia was reported to cause an ileum bezoar [14]. Although the paraclinical examinations did not describe a significant distal esophageal stricture, a previous diverticula could have been favored the bezoar development.

The radiological examination can frequently identify the bezoar and the associated conditions that predispose to bezoar development: epiphrenic diverticulum [15], strictures or motility disturbances like achalasia [16]. Regarding the submucosal pedunculated masses, barium studies are commonly used, but they have a low sensitivity to identify the pedicle [17–20]. The radiological correct diagnosis of fibrovascular polyp can usually be suggested by the presence of a smooth, sausage-like defect with a discrete bulbous tip [19]. The computer tomography or magnetic resonance imaging can identify in the most cases the exact site of origin of the pedicle of the fibrovascular polyps [19]. On computer tomography, the mesenchymal pedunculated tumors may appear as soft-tissue-attenuated lesions, with a paucity of fat, that expand the lumen of the esophagus [18].

The diagnostic challenge in our case continued in histological analysis. The presence of capillary structures, rare fibroblasts and collagen fibers were in favor of a connective tissue mass, most probable a fibrovascular polyp that suffered long process of calcification, argued by the numerous crystalline basophils deposits of minerals. The histological analysis excluded other mesenchymal tumors with intraluminal growth pattern (leiomyosarcoma, leiomyoma, gastrointestinal stromal tumor). The lack of adipocytes ruled out atypical lipomatous tumor that could mimick giant fibrovascular polyp of the esophagus [21].

For the differential diagnosis the most important raised question was if the vascular-connective tissue could have been found in a calcified alimentary bezoar? The connective tissue in this hypothesis must have been found at the periphery of the mass, but in our case it was homogeneous distributed into the calcified mass. For an ingested fibrous mass retained in a normal esophagus and than calcified there are no sufficient clinical and physiological arguments.

A serious contra-argument for a fibrovascular polyp is the lack of a pedicle (not identify at endoscopy, imagistic study or anatomo-pathological examination). But the presence of the vascular connective tissue described on histology is an important argument for its previous existence. In this case an auto-amputation process might be supposed.

The diverticula initially supposed to be the cause for the bezoar formation was actually secondary to mass compression. One month from the mass removal, the diverticula was not more identified at endoscopy, being another argument for an esophageal mesenchimal originating mass and a contraargument for a bezoar.

## Conclusion

Based on our challenging and difficult to diagnose case we proposed a natural uncommon evolution for an esophageal mesenchymal originating mass (most likely a fibrovascular polyp): auto-amputation and calcification.

## Consent

Written informed consent was obtained from the patient for publication of this Case report and any accompanying images. A copy of the written consent is available for review by the Editor of this journal.
